# Intracranial electrophysiological recordings on a swine model of mesial temporal lobe epilepsy

**DOI:** 10.3389/fneur.2023.1077702

**Published:** 2023-04-17

**Authors:** Fengjun Zhu, Hanwen Wang, Lin Li, Anatol Bragin, Dezhi Cao, Yuan Cheng

**Affiliations:** ^1^Department of Neurosurgery, Shenzhen Children’s Hospital, Shenzhen, Guangdong, China; ^2^Department of Neurosurgery, Second Affiliated Hospital of Chongqing Medical University, Chongqing, China; ^3^Department of Neurology, University of California Los Angeles, Los Angeles, CA, United States; ^4^Department of Biomedical Engineering, University of North Texas, Denton, TX, United States

**Keywords:** status epilepticus, kainic acid, swine model, mesial temporal lobe epilepsy, high-frequency oscillations

## Abstract

**Objective:**

To test the feasibility and reliability of intracranial electrophysiological recordings in an acute status epilepticus model on laboratory swine.

**Method:**

Intrahippocampal injection of kainic acid (KA) was performed on 17 male Bama pigs (*Sus scrofa domestica*) weighing between 25 and 35 kg. Two stereoelectroencephalography (SEEG) electrodes with a total of 16 channels were implanted bilaterally along the sensorimotor cortex to the hippocampus. Brain electrical activity was recorded 2 h daily for 9–28 days. Three KA dosages were tested to evaluate the quantities capable of evoking status epilepticus. Local field potentials (LFPs) were recorded and compared before and after the KA injection. We quantified the epileptic patterns, including the interictal spikes, seizures, and high-frequency oscillations (HFOs), up to 4 weeks after the KA injection. Test–retest reliability using intraclass correlation coefficients (ICCs) were performed on interictal HFO rates to evaluate the recording stability of this model.

**Results:**

The KA dosage test suggested that a 10 μl (1.0 μg/μl) intrahippocampal injection could successfully evoke status epilepticus lasting from 4 to 12 h. At this dosage, eight pigs (50% of total) had prolonged epileptic events (tonic-chronic seizures + interictal spikes *n* = 5, interictal spikes alone *n* = 3) in the later 4 weeks of the video-SEEG recording period. Four pigs (25% of total) had no epileptic activities, and another four (25%) had lost the cap or did not complete the experiments. Animals that showed epileptiform events were grouped as E + (*n* = 8) and the four animals showing no signs of epileptic events were grouped as E– (*n* = 4). A total of 46 electrophysiological seizures were captured in the 4-week post-KA period from 4 E + animals, with the earliest onset on day 9. The seizure durations ranged from 12 to 45 s. A significant increase of hippocampal HFOs rate (num/min) was observed in the E+ group during the post-KA period (weeks 1, 2,4, *p* < 0.05) compared to the baseline. But the E-showed no change or a decrease (in week 2, *p* = 0.43) compared to their baseline rate. The between-group comparison showed much higher HFO rates in E + vs. E – (*F* = 35, *p* < 0.01). The high ICC value [ICC (1, *k*) = 0.81, *p* < 0.05] quantified from the HFO rate suggested that this model had a stable measurement of HFOs during the four-week post-KA periods.

**Significance:**

This study measured intracranial electrophysiological activity in a swine model of KA-induced mesial temporal lobe epilepsy (mTLE). Using the clinical SEEG electrode, we distinguished abnormal EEG patterns in the swine brain. The high test–retest reliability of HFO rates in the post-KA period suggests the utility of this model for studying mechanisms of epileptogenesis. The use of swine may provide satisfactory translational value for clinical epilepsy research.

## 1. Introduction

Epilepsy is a chronic neurological disorder that affects 65 million people worldwide, characterized by the spontaneous occurrence of seizures (1). Current treatments fail to control epileptic seizures in 40% of patients and no preventative treatment is available (2). The acute insulting, chemical induction to gene-knockout, animal models of epilepsy provide an opportunity to study the latent period of this disease (3).

Animal models, such as rodents, are used to investigate the mechanisms and applications of epileptogenesis (4, 5). Rodents are inexpensive, well-studied, and can be genetically modified to produce a variety of genotypic backgrounds. However, the rodent brain greatly differs from the human brain in size, architecture, and composition; this limits the successful translation of information to human epilepsy treatment (6). Better translational science is needed to aid the development of therapies and improve the success rate of clinical trials (7).

Pigs are large animals of particular interest in neurological research. The porcine brain is a good alternative to the human brain and it displays higher connectivity and complexity in the cerebral cortex (8). The swine model now predominates in studies involving the treatment of neurological diseases (6). Due to the high proportion of white matter in the brain, the pig model is superior to small animals in investigating the pathophysiology of injury progression in traumatic brain injury (TBI), especially regarding the susceptibility of white matter to both focal and diffuse TBI (9). The pig brain is also superior to small animals for studying Huntington’s disease. Mice models do not sufficiently recapitulate the pathology seen in humans. They lack the overt and striking neurodegeneration pattern and have a poor resemblance in pathologic regions and circuits (10, 11). However, few studies have investigated pig epilepsy models. The most studied epilepsy model in pigs is the chemical seizure induction with penicillin-triggered epileptogenic activity (12–17). Penicillin injection results in a focal reduction of GABA-dependent inhibition leading to an increase in excitatory cortical afferents which triggers epileptiform bursts (18). A more-widely accessed model, the kainic acid (KA) (19), has not been tested in the porcine brain. Because of the more specific expression of KA receptors in the hippocampal CA3 areas, this model shows high efficiency in producing a region-specific seizure onset zone and simulating mesial temporal lobe epilepsy (mTLE) (19).

Other benefits of studying the intrahippocampal KA-induced mTLE include the similar lateral sizes of porcine and human brains and the feasibility of implanting a full-sized stereoelectroencephalography (SEEG) electrode (20). Swine (30–35 kg) were successfully implanted with subdural grid electrodes and used as a model for epilepsy surgical planning (15, 21). Previous studies have successfully measured epileptiform activity and seizures, but none have measured pathological high-frequency oscillations (pHFOs). It should be noted that pHFOs are the most prevalent biomarkers for evaluating epileptogenesis and potential candidates for epilepsy surgery (22, 23). Pathological HFOs were first discovered in the rodent model of mTLE (24–29) and their existence was confirmed in human patients with the same disease (24, 30, 31). Many studies have demonstrated that resection of the areas with the onset of the pHFO fast band helps to improve the surgical outcome in mTLE (30, 31). The study of pHFOs measured by full-size clinical SEEG electrode in a porcine model provides unique data on epileptogenesis, overcomes limitations in assessing data from human subjects in the latent period, and may be valuable in guiding the development of surgical techniques and antiepileptogenic therapies.

In this study, we explored the proper dosage of kainic acid required to induce status epilepticus and later development of mTLE in 25–35 kg laboratory swine. To achieve the translational value, we implanted full-size clinical SEEG electrodes into the porcine brain and studied the stability of neurophysiological patterns during status epilepticus and epileptogenesis. Considering the similarity of the porcine and human brains, our prediction was that signature pathological patterns, such as acute epileptic seizures and interictal epileptiform discharges (IEDs) would occur after KA injection (32–34). HFOs are prominent biomarkers of epileptogenesis and will show differences between the baseline period and epileptogenesis (24). To our knowledge, this is the first study to use laboratory swine to evaluate the latent period of epilepsy. We aim to provide a feasible option for translation study and improve the success rate of future clinical trials.

## 2. Materials and methods

### 2.1. Protocol

We used 2-to 3-month-old (*n* = 16) male Bama pigs (Taihe Biotechnology Co., Ltd.^®^) with weights ranging from 25 to 35 kg. The pigs were screened to ensure that they passed animal quarantine requirements and they were then housed in temperature-and humidity-controlled rooms during the study. SEEG electrodes were implanted under animal anesthesia followed by a confirmation of a structure MRI scan for the electrode location. The baseline recordings were conducted 2 days after implantation. KA doses were injected after the completion of baseline recording. Video monitoring was conducted 7/24 until the study was completed. EEG recordings were performed 2 h per day for 9–28 days, depending on the animal health and electrode cap endurance. After the experiments were completed, animals were euthanized. All of the experimental procedures followed the regulations of the Institutional Animal Care and Use Committee (IACUC) of Shenzhen Children’s Hospital.

### 2.2. Experimental procedure

#### 2.2.1. Electrode implantation

**Figure 1:**
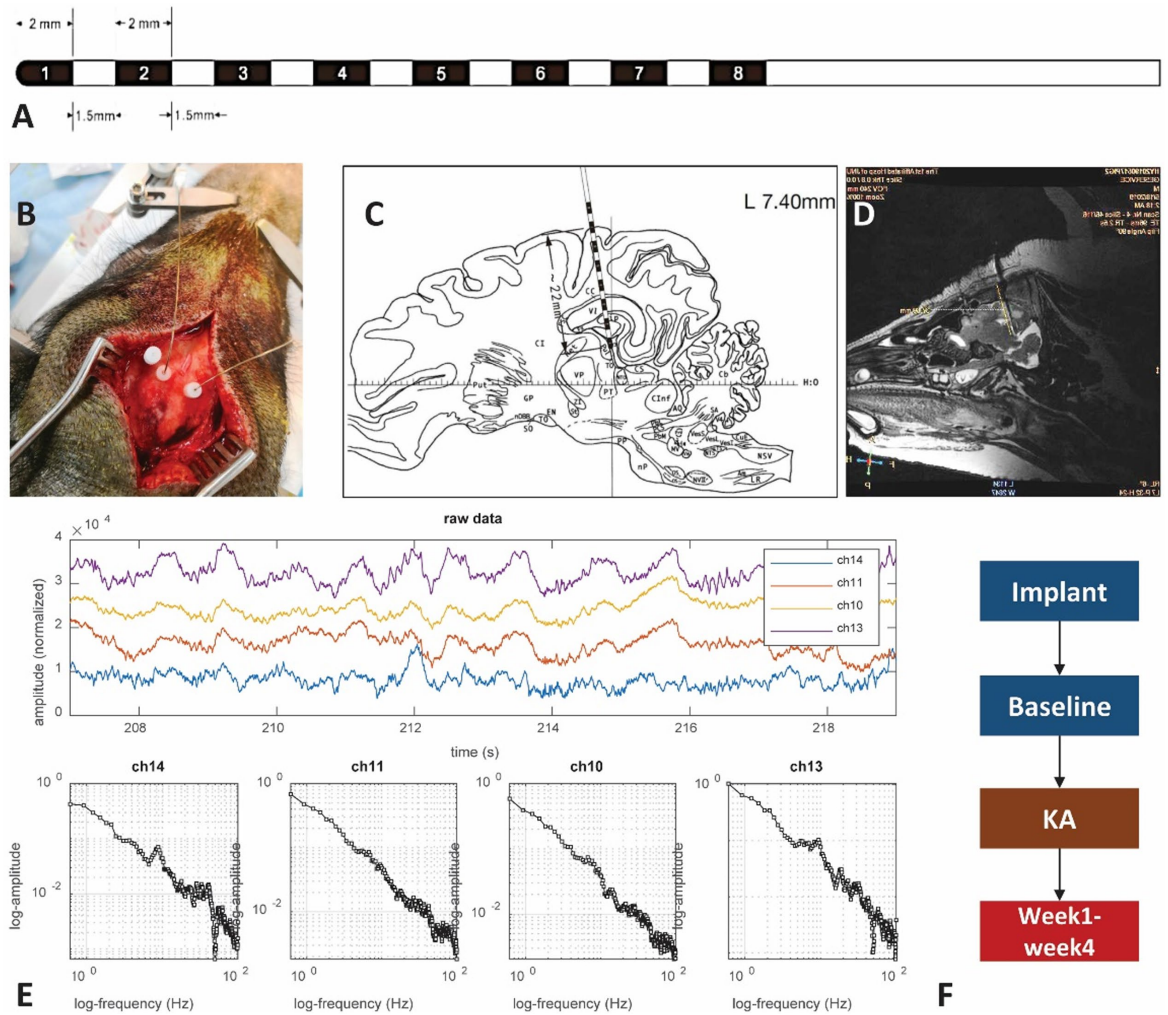
The experimental setup. **(A)** Illustration of the SEEG macroelectrode: the contacts on the electrode are labeled by black color and numbers. The contact is 2 mm in length with a gap of 1.5 mm between contacts and a dimension of 0.78 mm. **(B)** Photo showing the electrode implantation surgery. Hole marked by the letter “A” is preserved for KA injection. The other two dots connected with wires are electrodes. **(C)** Brain atlas of the electrode implantation. The distance from the superficial layer is about 22 mm, covering the sensorimotor cortex and hippocampus. Most of the time only the 6 contacts in the front are within the brain, with the corresponding channels 1–6 (left side) and 9–14 (right side). The contacts within the hippocampus area should be either from contacts 2–3 in the left or 9–11 from the right hemisphere. **(D)** MRI scan of the pig brain. The MRI scan has been used to verify the location of the SEEG electrode. **(E)** Examples of brain electrical activity (upper plots) and the results of multi-scale 1/f statistical behavioral analysis in the randomly selected epochs. The data indicate a clear near-linear decrease of log power with increasing log frequency from 0.5 to 100 Hz. The 50 Hz notch filter effect is visible in the channels that successfully reached the deep brain tissue. **(F)** The experimental protocols.

Two MRI-compatible SEEG electrodes were used for the implantation procedure. Each electrode contains 8 contacts from channels 1–8. The tip of the electrode has a diameter of 0.78 mm. The depth of each contact was 2.0 mm, with an interval of 1.5 mm between two adjacent contacts (Figure 1A). The electrode contacts were made of a smooth platinum-rhodium (Pt-Rh) alloy with platinum-iridium (Pt-Ir) wire soldered to the inner wall, with an impedance of 6–7 kOms. As guided by the atlas (35), electrodes were implanted bilaterally to the coordinates: [AP] = −5 mm, [ML] = 7 mm, [DV] = 22 mm. Glue was used to attach the electrodes to the skull. After surgery, animals were returned to their cages to rest. An MRI scan on the second day after implantation confirmed the precise location of the electrodes.

#### 2.2.2. Kainic acid injection and dosage testing

A KA injection was given on day 7 after electrode implantation (Figure 1F). Before conducting the KA protocol, a brief dosage test that lasted 24 h was performed to estimate the proper amount of KA to inject. Since there is no reference to the KA-swine model, we referred to the KA-treatment guidance for rats (36). Three different dosages: 5 μl (1.0 μg/μl), 10 μl (1.0 μg/μl), and 15 μl (1.0 μg/μl) were tested in three control pigs to find the effective dose for the induction of status epilepticus while minimizing body impairment. A craniotomy at [AP] = −3 mm, [ML] = 15 mm, [DV] = 25 mm was performed (Figure 1B). KA was injected into the hippocampus CA3 area within 10 min with a sterilized microsyringe. The tip of the microsyringe stayed within the brain for 2 min before being removed.

#### 2.2.3. Electroencephalography recordings

LFPs, at the sampling rate of 4,000 Hz, were acquired by a clinical EEG device (Nihon Kohden^®^). Recordings started on day 2, lasted for 4 days for the baseline and 7–23 days for the post-KA Period. The 16-channel recordings were performed at the same time from two hemispheres, with channels 1–8 in the left brain and channels 9–16 on the right side (Figure 1C). A mixed anesthetic protocol was applied. At the beginning of the anesthesia, an intramuscular injection of 0.02 mg/Kg atropine sulphate (AS) was performed. After 15 min, intramuscular injection of 2.5 mg/kg Zoletil^®^50 and 2 mg/kg xylazine hydrochloride (XH) was performed. When the swine entered a state of mild anesthesia (about 30 min) the EEG recording started and lasted for 2–4 h during each recording day. The local field potentials (LFPs) were recorded for 2 days as a baseline period. Following the KA injection, the pigs were returned to their holding cages and allowed to recover for 4–6 days. Then the post-KA recording started and was performed for 2 h daily for a total of 4 weeks. Data quality was reviewed by screening raw data combined with the results of multi-scale 1/f statistical behavioral analysis in the randomly selected epochs (Figure 1E). The data with a clear near-linear decrease of log power with increasing log frequency from 0.5 to 100 Hz was considered as an indication of good recording (37).

#### 2.2.4. MRI scan

MRI scans were performed with the GE Discovery MR750 3.5 T MRI Scanner (GE Medical^®^) twice during the entire experiment. One 3D BRAVO T1 scan (512 × 512 × 116, TR = 9.2 ms, TE = 3.7 ms, TI = 450 ms, 0.43 × 0.43 × 1 mm, FOV = 220 × 220 mm) and a T2 scan (512 × 512 × 116, TR = 2,500 ms, TE = 96.4 ms, 0.47 × 0.47 × 0.8 mm, FOV = 240 × 240 mm) were performed. An MRI scan was performed on each pig to confirm the electrode implantation site (Figure 1D). A 22 mm length (from the tip of the electrode) was inserted into the brain, which included channels 1–8 in the left hemisphere and channels 9–16 in the right hemisphere. The Figure 1D also displayed the contacts of the electrode with hippocampus areas in channels 1–3 and channels 9–11 for the left and right hippocampus, respectively.

### 2.3. Data analysis

#### 2.3.1. Data review and preprocessing

The EEG data were first converted to the European Data Format (EDF). The recordings were reviewed by two experts in EDFbrowser^1^ and EEG-Lab (38). Motion artifacts and bad channels were manually removed from the recording.

#### 2.3.2. Multi-taper analysis

A multi-taper spectral analysis (39) was used to distinguish the sleep stage under mild anesthesia. The procedures of the multi-taper technique had three sub-steps. The first step was to create time bins. Since the high-frequency resolution was preferred in this experiment, the length of the time window was set at 30 s and the step size was set at 1 s to achieve a high overlapping rate of two consecutive time bins. The second step was to get the power vector at the *i*th time slot $P_{i}=\frac{1}{m}\overset{m}{\underset{j=1}{\sum }}z.*\bar{z}$ , in which m denotes the number of different tapers, $z=2*fft\left(d_{i}*T_{j}\right)/n$ and $\bar{z}$ is the complex conjugate of z . Specifically, $d_{i}$ is the raw data of the corresponding *i*th time window, $T_{j}$ is the *j*th normalized orthogonal taper vector, and n is the number of points in each time window. The last step was to generate the time-frequency matrix P . The vector $P_{i}$ is the *i*th column of P .

#### 2.3.3. Interictal epileptiform discharges detection

Detection of interictal epileptiform discharges (IEDs) was performed using the following steps: (i) band-pass filtering was performed at 25–80 Hz to account for the wider waveforms of IEDs observed; (ii) thresholds were adjusted to two times above baseline; and (iii) IEDs occurring within 1 s of another IED were excluded to prevent over-correlation due to a run of IEDs. Detected IEDs had waveforms consistent with typical interictal spikes and/or sharp waves.

#### 2.3.4. Seizure detection

A combined approach was used for seizure detection. The electrophysiological seizures were detected automatically by an unsupervised, multi-threshold approach (40). Specifically, a comprehensive method combined the complex wavelet transform (CWT) with multi-layer thresholding and was implemented for both noise reduction and seizure detection. The detected seizure epochs were confirmed and manually marked by the experts. The behavioral seizures were evaluated by the video recordings. The seizure detection was performed by using a dynamic threshold method. The data were first evaluated for a seizure band (1–25 Hz) threshold. The seizure activity energy was computed and a dynamic threshold of two standard deviations was applied to detect the lower and upper boundaries of seizure energy. Along with the threshold, the epileptic event peaks were also detected and a frequency histogram was computed (40).

#### 2.3.5. High-frequency oscillation detection

EEG datasets were loaded into MATLAB which performed an HFO auto detection algorithm. Details of this method were described previously (41). HFO events were then extracted and false-positive events were rejected artificially with Ripplelab (42). The automatic HFO detections were performed in the RippleLab toolbox through MATLAB 2016a. Specifically, EEG data were: (1) bandpass filtered 100–500 Hz to identify high-frequency EEG events; (2) calculated for the root mean square (RMS; 3-ms window) of the band-pass signal; and (3) evaluated by successive RMS values greater than 5 SD above the overall mean RMS value and with a minimum of 6 ms in duration between the onset and offset boundaries. HFO events were subjected to the additional criterion of containing a minimum of 6 peaks that are greater than 3 SD above the mean value of the rectified band-pass signal. All of the automatically detected HFOs were visually examined and selected by two experienced investigators (Dr. Li and Dr. Bragin). HFO data that were within the same animal group (E –, E +) were combined into larger files and the HFO events were detected based on the onset of the oscillations (43).

#### 2.3.6. Statistics

Statistical analysis was performed using one-way analysis of variance (ANOVA) for comparison of the total HFO rate among channels. Two-sample t tests were performed to test the significance of the difference between baseline recordings and KA-lesioned data. Differences between groups were calculated using ANOVA (Bonferroni correction). Error bars represent the standard error of the mean (SEM). The significance level was *p* < 0.05.

To validate the result stability, we quantified the test–retest reliability using the HFO occurrence rate (num/min) during the four-week post-KA period. We considered each week as one test so that week 1–week 4 constituted four repeated measurements. The intraclass correlation coefficient (ICC) was a common parameter used to estimate measurement reliabilities (44–46). We applied the one-way random effect model of ICC (1, *k*) for the HFO rates to assess the reliability of the repeated measurement (44–46). We considered the animals included more than one variable (animal groups and weeks), which were fitted to the ICC (1, *k*) model (44, 47). For the evaluation, we followed the guidance of clinical significance as the reliability and considered poor, fair, good, and excellent when ICC < 0.40, 0.40 < ICC < 0.59, 0.60 < ICC < 0.74, and 0.75 < ICC < 1.00, respectively (47, 48).

## 3. Results

### 3.1. Successfully constructed KA-induced status epilepticus in laboratory swine

Of the three pigs that underwent dosage testing (Table 1), all of the quantities of KA triggered interictal spikes after 30 min of injection. For the 5 μl dose, the acute epileptic events only occurred in the first 2 h but there were no seizure activities for the first 24 h. The 10 μl KA dose produced approximately 6 h of interictal spikes and 11 acute seizures within the first 24 h. The 20 μl dose showed similar numbers of interictal spikes and triggered more than 50 acute seizures within 24 h. However, the pig exposed to a 20 μl dose died on the second day. Based on this, we chose 10 μl for the KA injection experiment in consideration of the survival rate and the effectiveness of the mTLE model.

**Table 1:** Responses of three kainic acid (KA) doses in 0–24 h after injection.

Animal ID	KA dose	Interictal spikes	Number of acute seizures	Survive
01	5 μl (1.0 μg/μl)	30 min after injection, total < 1 h w/24 h.	None w/24 h	Yes
02	10 μl (1.0 μg/μl)	30 min after injection, total 4 h w/24 h.	11 w/24 h	Yes
03	20 μl (1.0 μg/μl)	30 min after injection, total 5 h w/24 h.	>30 w/24 h	None

For the 16 animals exposed to the 10 μl KA injection, 12 animals successfully completed the 4-week experiments, two animals died in the first week following injection and two animals lost their cap and were excluded from the dataset. Chronic-tonic seizures were captured in four pigs in weeks 2–4. Besides the four pigs with seizure occurrence, interictal spikes or short bursts of seizure-like events were observed in another four pigs. These eight animals were classified as the epileptic group (E +, *n* = 8) in this study. The remaining four animals showed no epileptic-like activities during the experimental period and were classified as the animals do not have epilepsy group (E–, *n* = 4).

### 3.2. Electrophysiological patterns in baseline and epileptogenesis

**Figure 2:**
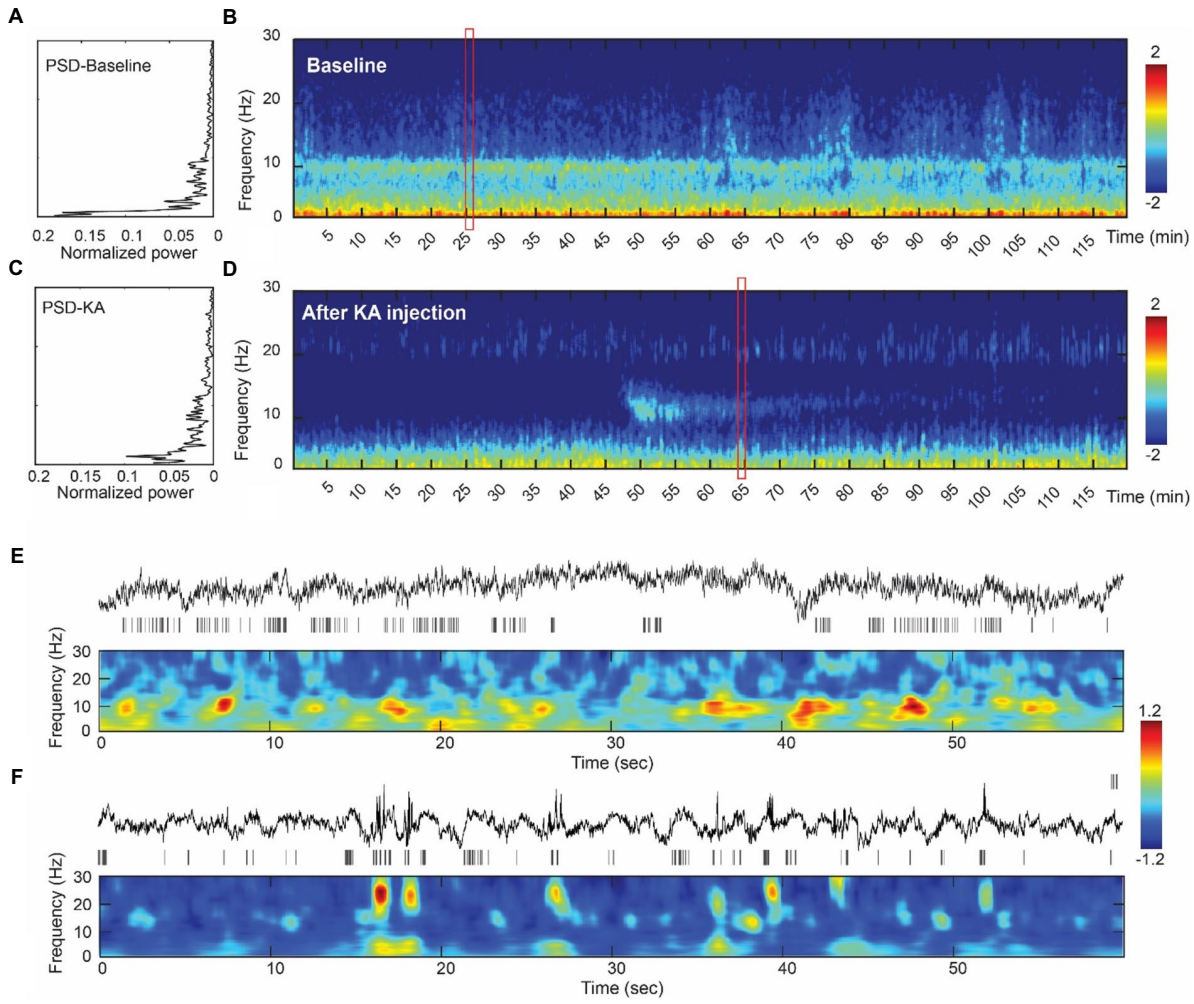
Assessment of the intracranial recordings before and after KA injection. **(A)** The analysis of power spectrum from the baseline recording. **(B)** An illustration of multi-tapper analysis from the 2-h epoch of baseline data. A clear eye-closed alpha band occurs in both PSD and multi-tapper plots. **(C)** Power spectrum plot from the animal after the KA-lesion (from week1). **(D)** The multi-tapper analysis from the 2-h epoch of the data after KA-lesion (from week1). **(E)** (Top-bottom) Demonstration of the 60 s local field potentials (LFPs) and the time-frequency decomposition (0–30 Hz). Data were obtained in the redbox shown in **(B)**. **(F)** Similar plots as Figure E, but obtained from the data after KA injection (redbox in D). Compared to the baseline, data from the post-KA period showed a suppression of the lower frequency band (delta wave, 0–3 Hz) and a decrease of the alpha band (10 Hz), but an increase of the spindle-like activity (see the bursts > 20 Hz).

Electrical activity was recorded under mild xylazine hydrochloride anesthesia. Power spectrum density (PSD) analysis suggested a peak frequency at 4 Hz and 10 Hz in baseline recording (Figure 2A), which represents the delta wave and alpha wave in EEG. They are related to the non-rapid eye movement (NREM) sleeping stage (49, 50). Compared to the baseline, PSD analysis of 2 h LFPs from the post-KA period indicated a decrease of delta and alpha power and an appearance of the 18–20 Hz peak in the hippocampus (Figure 2C). The multi-taper analysis also revealed differences in time-frequency patterns of the two experimental conditions (Figures 2B,D). The alpha wave disappeared in the data after KA injection.

### 3.3. Epilepsy features identification

**Figure 3:**
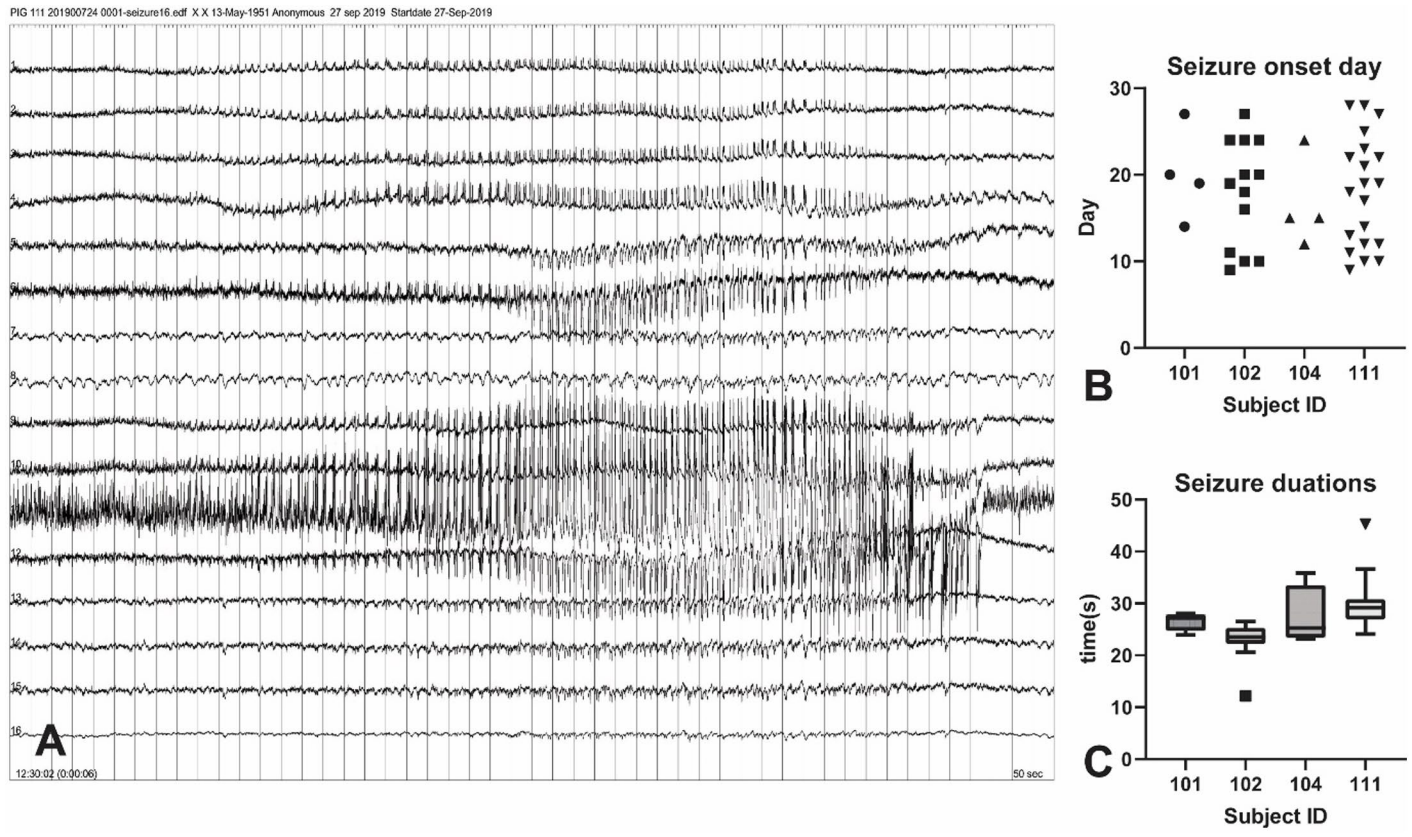
Seizure events in the KA-pig model of epilepsy. **(A)** Screenshot of a tonic–chonic electrophysiological seizures onset in an animal after KA injection. The onset is located in channel 11, later confirmed to be the right hippocampus. A total of 46 electrophysiological seizures were captured in the 4-week post-KA period from 4 animals (25% of the total). **(B)** The analysis of the seizure onset day. The earliest chronic seizure onset was observed in animal 111, on day 9 (shown in Figure A), and the last one was on day 27. The first seizure onset day is from 9–14 in these animals. **(C)** Seizure durations from all recorded 46 seizures in 4 pigs. The seizure duration ranged from 22 s to 35 s.

A total of 46 tonic-chronic electrophysiological seizures were identified in the 4-week post-KA period from four pigs (Figure 3). The earliest seizure onset was at day 9 (in subject 102) and the latest was recorded at day 28 (the last day of measurement). The average seizure onset day was (18 ± 5.96), and the number of onsets was 4, 13, 4, and 20 in each animal. The seizure duration ranged from 12 to 45 s (mean = 27.21 ± 5.20).

### 3.4. Occurrence of HFOs in the baseline and post-KA period

**Figure 4:**
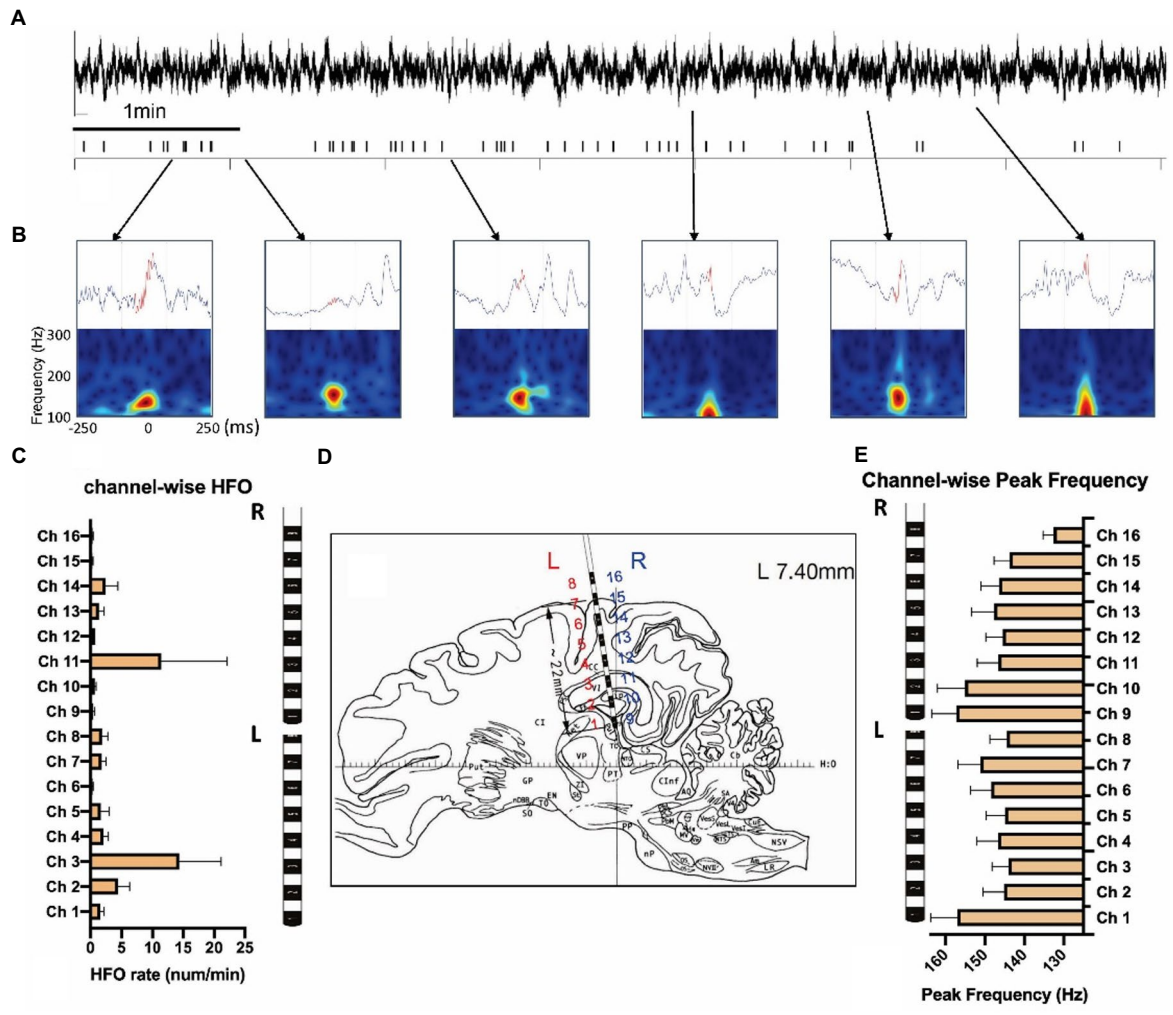
Measurement of high-frequency oscillations after the KA injection. **(A)** LFPs and the high-frequency oscillations (HFOs) of 10 min KA-lesioned EEG signals. **(B)** Illustration of HFO events: local field potentials (upper) and time-frequency plots (bottom). **(C)** Channel-wise HFO rate analyzed from the week1 data of 8 animals. **(D)** The brain atlas showing the electrode implantation. The electrode contacts 1–8 are on the left (L), and contacts 9–16 are located on the right (R) side of the brain. **(E)** Channel-wise peak frequency of the same group.

We quantified 812 HFOs from the sensorimotor and hippocampal channels. All HFO events were manually verified in the data with a 500 ms LFP and time-frequency plot centered with HFO peaks after computational detection (see Figures 4A,B and Supplementary Figure 1 for examples). HFO rates were calculated on each channel, with the highest rate displayed in ipsilateral hippocampus (14.36 ± 6.76/min) and contralateral hippocampus (11.47 ± 7.06/min; Figures 4C,D). Though the HFO rates were much larger in these two channels (*F* = 2.49, *p* = 0.002) they were statistically similar to each other (*p* = 0.29). Peak frequencies ranged from 132 to 157 Hz, with an average of 147.3 ± 20.45 Hz. We found no significant difference of peak frequencies (*F* = 1.30, *p* = 0.20) between each channel (Figure 4E).

**Figure 5:**
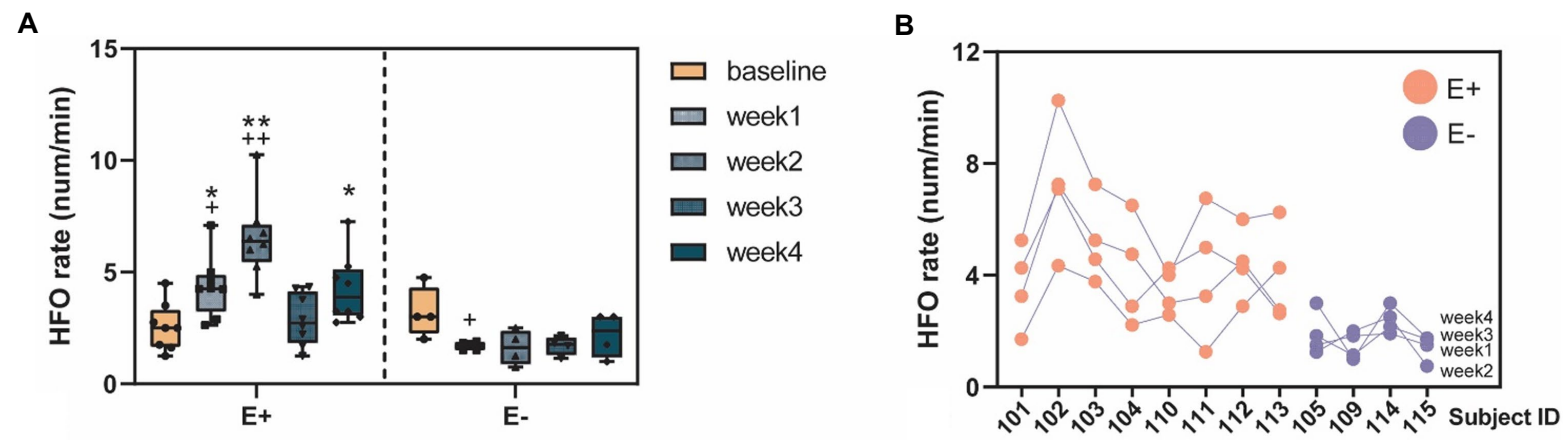
High-frequency oscillation (HFO) occurrences before and after KA injection. **(A)** The HFO rates (num/min) quantified in the E+ animals (n = 9) that showed clear epileptic events (IEDs or seizure) and the E– animals (*n* = 4) that did not show any signs of epileptiform events after the KA injection. The E + animals had an increase of HFO rates in the week1–4 post-KA period, compared to the baseline while the E– animals showed no or a small decrease of the HFO rates (week1 vs. baseline, *p* = 0.43). The E + animals also showed much larger HFO rates in week1, 2, and 4, compared to the E– group. **(B)** Stability of the individuals weekly HFO rates. Each blob represents the HFO rate in 1 week. Colors indicate the animal group: red = E + and blue = E –. ^*^p < 0.05; ^**^p < 0.001 between group comparison: E + vs. E –; ^+^ *p* < 0.05; ^++^ *p* < 0.001 within group comparison: baseline vs. week 1–4.

We studied the within-and between-group differences of HFO rates corresponding to different experimental periods (baseline vs. post-KA). In the within-group comparison, there were significant increases of HFO rates in the E + group (*F* = 10, *p* < 0.001) in the post-KA period compared to the baseline, specifically observed in week1 (*p* = 0.47) and week2 (*p* < 0.001). However, there were no significant differences in HFO rates in the E-group (*F* = 2.8, *p* = 0.065), though there was a slight decrease in the HFO rate in week 1 (*p* = 0.43). For the between-group comparison, we found E+ > E– in week1 (*p* = 0.005), week2 (*p* < 0.001), and week4 (*p* = 0.48). The HFO rates of E + and E – in the baseline were similar (*p* = 0.93; Figure 5A).

The stability of the HFO measurements was assessed daily from the 2 h recordings. HFOs from both hippocampus and sensorimotor cortex were compared between baseline and during epileptogenesis. In total, the HFO rates were significantly larger in the post-KA period compared to baseline (*p* = 0.39). Among the time epochs, most of the epochs showed significantly larger HFO rates in epileptogenesis than baseline (Figure 5A). There was no difference in the HFO rates in total and in each epoch in the sensorimotor cortex (except for some channels where no HFOs were detected). Analysis of peak frequency revealed no differences between baseline and in epileptogenesis except for channel 6 (*p* = 0.011).

### 3.5. Test–retest reliability of the HFOs in the 4-week post-KA recording period

The ICC (1, *k*) = 0.81 [LB = 0.54, UB = 0.94, *F* = 5.14, *p* < 0.001] was computed from the weekly HFO rate data, suggesting excellent (ICC > 0.8) test–retest reliability of results. These data were obtained by grouping all of the subjects, including both E + and E – (Supplementary Table S1 and Figure 5B). We also studied the data by separating the E + and E – groups, and found fair reliability [ICC (1, *k*) = 0.49] in the E + group and fair reliability [ICC (1, *k*) = 0.59] in the E– group.

## 4. Discussion

We evaluated the KA-induced mTLE model on laboratory swine. With two SEEG electrodes, we studied the ictal and interictal electrophysiological patterns, including electrophysiological seizures, interictal spikes, and HFOs, during a 4-week experimental period. Using an optimal chemo-convulsant dosage, the model showed an efficient initial precipitant injury afflicting the hippocampus, and then a latent period followed by the occurrence of spontaneous seizures (in 25% of animals) and interictal spikes (in 56% of animals). For the animals that developed chronic epileptic activities, a significant increase of hippocampal HFO rates occurred in the post-KA period, compared to the animals without epilepsy. This study introduces a KA-induced model in swine. Swine brains are similar to human brains in size and morphology; thus, they are appropriate for use in future translational studies on epilepsy.

### 4.1. The swine model of mTLE

A limitation in the experimental design of this study was the lack of prior knowledge, especially for the swine model of mTLE. Most prior studies on pigs have used subcortical penicillin (PCN) injections, which acted as a γ -aminobutyric acid (GABA)_A_ antagonist. The results demonstrated effective physiological changes in vital signs that occurred during convulsive activities in the treated pigs (13, 51–54). Various dosages of penicillin were used ranging from low (10 μl; (53)) to high (100 μl; (52)). However, even with the relatively larger size of the pig brain, a lower dose of PCN (10 μl) is sufficient to produce focal epileptiform activities and solid clinical seizure activities from grade II to IV (15).

Compared with the subcortical epilepsy model using PCN, the KA-model in the pig hippocampus produces an efficient local lesion with the chronic manifestation of spontaneous seizures that histopathological results showed as mTLE (3). KA-injection is a mature model in rodents. KA can preferentially destroy hippocampal pyramidal cells and trigger neurodegeneration in the hippocampus, but the affected region varies with dosage and the hippocampus is very sensitive to the KA level. Intraventricular injection of KA (at 0.5 nmol) in Sprague–Dawley rats caused pyramidal cell degeneration in CA3 at the rostral pole of the hippocampus whereas higher doses (0.8 μg) caused neuronal loss in more caudal regions of the hippocampus (19). Doses higher than 0.8 μg induced neurodegeneration in CA1 and CA2 (19). In rats, intrahippocampal administration of KA at a dosage between 0.4 and 2.0 μg is usually effective in inducing a convulsive status epilepticus after 5–60 min following the injection. Considering the PCN-swine model and the KA-model of rodents, we initially used a relatively narrow dosage range of 5, 10, and 20 μl. Initial testing with three doses indicated that 10 μl is close to the optimal concentration for the KA-model in swine (Table 1). All three initial cases showed epileptic activities but only case2 (10 μl) and case3 (20 μl) evoked tonic–chronic seizures. Case3 exposed to a 20 μl KA injection had a severe neuromuscular blockade and vigorous convulsions lasting more than 5 h and died within a short time. The remaining animals tested with 10 μl had a high (87.5%, 14/16) survival rate. These data were similar to results reported using the rodent model (with a mortality rate of 12%) (55–57).

### 4.2. Ictal and interictal electrophysiological patterns

Interictal patterns during slow wave sleep have been widely reported in animal epilepsy models. Similarly, an obvious change in frequency components was observed pre- and post-KA injection. There were 4 and 10 Hz frequency peaks on the power spectrum in baseline recordings. However, the 10 Hz frequency peak vanished after KA injection and a frequency peak of 20 Hz appeared within a short period (Figure 2). While the power spectrum only displayed the overall compositions of frequency, time-frequency analysis with the multi-taper method were further involved to display the frequency distribution along 2-h interval recordings. A 10 Hz frequency component, also known as sleep spindles, was usually seen in stage 2 of NREM sleep and plays an important role in memory consolidation (58). The disappearance of sleep spindles after status epilepticus may indicate the occurrence of pathological changes in cerebral areas, and the post-epilepsy memory injury related to human patients. Sleep disturbance is also a common sign in epilepsy patients (59, 60).

Despite the KA-evoked acute epileptic responses, we captured a total of 46 electrographic seizures from four animals from 9 to 28 days. As the first onset was more than 7 days after the KA injection, and each animal had more than one onset, we considered these events to be tonic-chronic seizures. Compared to the rodent intrahippocampal KA model, we found the first onset of the chronic seizures was a 9 days, which is later than rats (5 days) (19). The majority (66%, 8/12) of the tested pigs showed clear signs of epileptogenesis, and this rate was higher than a previous study of the intrahippocampal KA rat model (54%) (41).

### 4.3. High-frequency oscillation occurrence in the mTLE model of laboratory swine

The HFOs occur in various brain areas, in both normal and pathological conditions (28, 61). In previous studies using a rodent KA-model of mTLE, there was an increase of ripple, and an appearance of fast ripple activities (41, 62, 63) during the latent period of epileptogenesis. With two 16-channel SEEG electrodes, each with a contact area of 2 mm, we successfully detected HFO activities in the swine hippocampus in the baseline and after KA injection. A noticeable difference was the significant increase of the HFO rate after KA injection, compared to the baseline period. Consistent with the rodent model, the increase of HFO rates indicated changes in the local circuits based on the formation in pathologically interconnected neuron clusters (PIN-cluster) (64). We did not find any fast ripple activities; all of the HFO events were in the 100–200 Hz frequency range of ripple. This may be because the 2 mm contact area in the SEEG channels is too large to collect the local activates yield with fast ripples.

The HFO events in baseline displayed an evenly distributed pattern, while after KA injection, HFO distributions were mostly constrained within interictal spikes. Previous studies found that the pathological high frequency oscillations (pHFOs) often co-occur with interictal spikes (32, 65, 66). Interictal spikes (and co-occurring pHFOs) may disrupt memory processing (67). Most studies have demonstrated that interictal events disrupt memory consolidation processes, as interictal spikes have negative impacts when they occur during retrieval versus encoding phases of memory tasks (68). During sleep, interictal spikes inappropriately initiate spindle activity in the cortex and disrupt memory consolidation processes that engage cortical areas. The highest HFO rate was detected from the hippocampus area, which is similar to reports on mTLE patients (69). A high test–retest reliability of HFO rates was observed in the post KA period. The weekly stability suggested a reliable measurement of HFOs as critical biomarkers of epileptogenesis (25, 27, 31). There was no change or decrease of the HFO rates in the pigs do not have epilepsy (the E– animals). This is different from findings using the rodent model (41), where both E + and E – rats showed an increase of HFOs in the post-KA phase during the entire 5-week experimental period.

### 4.4. Pitfalls and future directions

The animal phenotyping for the E + and E – groups was based on the occurrence of recurrent seizures observed in the post-KA period as well as the interictal spikes. Although interictal spikes normally indicate epileptic events, they are not a consistent biomarker for the development of chronic epilepsy. Other severe neurological conditions, such as stroke also shared the similar patterns of interictal spikes (70, 71). Our study duration was 4 weeks and omitted animals that had only been observed with IEDs but not seizures. However, the four animals classified as the E– group may have a chance to develop spontaneous epilepsy. This pitfall could be addressed by increasing the experimental period but doing so would increase the maintenance issue as the pigs gained weight and increased in size. The daily movement behavior of animals is also a challenge for the endurance of the EEG caps. For simplicity, this study only used two SEEG electrodes. The use of this model could be further investigated on a large scale by planning the subdural EEG and more intracranial EEGs. Overall, this study suggests that a KA-induced swine model is useful for studying the mechanisms and translational applications of epilepsy.

## Data availability statement

The original contributions presented in the study are included in the article/Supplementary material, further inquiries can be directed to the corresponding authors.

## Ethics statement

The animal study was reviewed and approved by Shenzhen Children’s Hospitals.

## Author contributions

FZ completed the animal experiment. FZ and HW performed the data analysis and wrote the draft. LL and AB gave advice on the data analysis and experimental design. DC and YC initialized this research. All authors contributed to the article and approved the submitted version.

## Funding

This study was supported by the Shenzhen Children’s Hospital Epilepsy Center Research Funding (DC), Shenzhen Children’s Hospital-UCLA Visiting Scholar Program (FZ & DC), and The National Institute of Health 1R16-NS131108-01 (LL).

## Conflict of interest

The authors declare that the research was conducted in the absence of any commercial or financial relationships that could be construed as a potential conflict of interest.

## Publisher’s note

All claims expressed in this article are solely those of the authors and do not necessarily represent those of their affiliated organizations, or those of the publisher, the editors and the reviewers. Any product that may be evaluated in this article, or claim that may be made by its manufacturer, is not guaranteed or endorsed by the publisher.

## Footnotes

^1^https://www.teuniz.net/edfbrowser/

## References

[1] Devinsky O Vezzani A O'Brien TJ Jette N Scheffer IE De Curtis M. Epilepsy. Nat Rev Dis Primers. (2018) 4:18024. doi: 10.1038/nrdp.2018.24 29722352

[2] Shorvon S Perucca E Engel J Jr. The Treatment of Epilepsy. New York, NY: John Wiley & Sons (2015).

[3] Kandratavicius L Balista PA Lopes-Aguiar C Ruggiero RN Umeoka EH Garcia-Cairasco N. Animal models of epilepsy: use and limitations. Neuropsychiatr Dis Treat. (2014) 10:1693–705. doi: 10.2147/NDT.S50371, PMID: 25228809 PMC4164293

[4] Rakhade SN Jensen FE. Epileptogenesis in the immature brain: emerging mechanisms. Nat Rev Neurol. (2009) 5:380. doi: 10.1038/nrneurol.2009.80 19578345 PMC2822660

[5] Pitkänen A Lukasiuk K. Mechanisms of epileptogenesis and potential treatment targets. Lancet Neurol. (2011) 10:173–86. doi: 10.1016/S1474-4422(10)70310-0, PMID: 21256455

[6] Hoffe B Holahan MR. The use of pigs as a translational model for studying neurodegenerative diseases. Front Physiol. (2019) 10:838. doi: 10.3389/fphys.2019.00838 31354509 PMC6635594

[7] Pitkänen A Kharatishvili I Karhunen H Lukasiuk K Immonen R Nairismägi J. Epileptogenesis in experimental models. Epilepsia. (2007) 48:13–20. doi: 10.1111/j.1528-1167.2007.01063.x 17571349

[8] Lind NM Moustgaard A Jelsing J Vajta G Cumming P Hansen AK. The use of pigs in neuroscience: modeling brain disorders. Neurosci Biobehav Rev. (2007) 31:728–51. doi: 10.1016/j.neubiorev.2007.02.003, PMID: 17445892

[9] Kinder H Baker E West F. The pig as a preclinical traumatic brain injury model: current models, functional outcome measures, and translational detection strategies Neural Regen Res, (2019); 14:413–424. Review 30539807 10.4103/1673-5374.245334 PMC6334610

[10] Yan S Tu Z Liu Z Fan N Yang H Yang S. A huntingtin Knockin pig model recapitulates features of selective neurodegeneration in Huntington’s disease. Cells. (2018) 173:989–1002e13. doi: 10.1016/j.cell.2018.03.005 PMC5935586 29606351

[11] Ratna N Jain S. Huntington's disease pig model: squealing into the spotlight. Mov Disord. (2018) 33:1410–1. doi: 10.1002/mds.96, PMID: 30311975

[12] Terndrup TE Starr F Fordyce WE. A piglet model of status epilepticus: comparison of cardiorespiratory and metabolic changes with two methods of pentylenetetrazol administration. Ann Emerg Med. (1994) 23:470–9. doi: 10.1016/S0196-0644(94)70065-6, PMID: 8135421

[13] Leaming JM Terndrup TE Ognibene S. Glottal patency during experimental cortical seizures in piglets. Acad Emerg Med. (1999) 6:682–7. 10433526 10.1111/j.1553-2712.1999.tb00435.x

[14] Mäkiranta M Ruohonen J Suominen K Niinimäki J Sonkajärvi E Kiviniemi V. BOLD signal increase preceeds EEG spike activity—a dynamic penicillin induced focal epilepsy in deep anesthesia. NeuroImage. (2005) 27:715–24. doi: 10.1016/j.neuroimage.2005.05.025, PMID: 16006147

[15] Van Gompel JJ Bower MR Worrell GA Stead M Meier TR Goerss SJ. Swine model for translational research of invasive intracranial monitoring. Epilepsia. (2011) 52:e49–53. doi: 10.1111/j.1528-1167.2011.03096.x, PMID: 21627648 PMC3116097

[16] Van Gompel JJ Bower MR Worrell GA Stead M Chang SY Goerss SJ. Increased cortical extracellular adenosine correlates with seizure termination. Epilepsia. (2014) 55:233–44. doi: 10.1111/epi.12511, PMID: 24483230 PMC4104491

[17] Witkowska-Wrobel A Aristovich K Crawford A Perkins JD Holder D. Imaging of focal seizures with electrical impedance tomography and depth electrodes in real time. NeuroImage. (2021) 234:117972. doi: 10.1016/j.neuroimage.2021.117972 33757909 PMC8204270

[18] Fisher RS. Animal models of the epilepsies. Brain Res Rev. (1989) 14:245–78. doi: 10.1016/0165-0173(89)90003-9 2679941

[19] Lévesque M Avoli M. The kainic acid model of temporal lobe epilepsy. Neurosci Biobehav Rev. (2013) 37:2887–99. doi: 10.1016/j.neubiorev.2013.10.011, PMID: 24184743 PMC4878897

[20] Sauleau P Lapouble E Val-Laillet D Malbert C-H. The pig model in brain imaging and neurosurgery. Animal. (2009) 3:1138–51. doi: 10.1017/S1751731109004649, PMID: 22444844

[21] Bower MR Stead M Van Gompel JJ Bower RS Sulc V Asirvatham SJ. Intravenous recording of intracranial, broadband EEG. J Neurosci Methods. (2013) 214:21–6. doi: 10.1016/j.jneumeth.2012.12.027, PMID: 23313850 PMC3593671

[22] Staba RJ Bragin A. High-frequency oscillations and other electrophysiological biomarkers of epilepsy: underlying mechanisms. Biomark Med. (2011) 5:545–56. doi: 10.2217/bmm.11.72, PMID: 22003903 PMC3233380

[23] Milikovsky DZ Weissberg I Kamintsky L Lippmann K Schefenbauer O Frigerio F. Electrocorticographic dynamics as a novel biomarker in five models of Epileptogenesis. J Neurosci. (2017) 37:4450–61. doi: 10.1523/JNEUROSCI.2446-16.2017, PMID: 28330876 PMC6596657

[24] Bragin A Engel J Jr Wilson CL Fried I Buzsáki G. High-frequency oscillations in human brain. Hippocampus. (1999) 9:137–42. doi: 10.1002/(SICI)1098-1063(1999)9:2<137::AID-HIPO5>3.0.CO;2-0 10226774

[25] Frauscher B Bartolomei F Kobayashi K Cimbalnik J Vant Klooster MA Rampp S. High-frequency oscillations: the state of clinical research. Epilepsia. (2017) 58:1316–29. doi: 10.1111/epi.13829, PMID: 28666056 PMC5806699

[26] Jiruska P Alvarado-Rojas C Schevon CA Staba R Stacey W Wendling F. Update on the mechanisms and roles of high-frequency oscillations in seizures and epileptic disorders. Epilepsia. (2017) 58:1330–9. doi: 10.1111/epi.13830, PMID: 28681378 PMC5554080

[27] Levesque M Shiri Z Chen LY Avoli M. High-frequency oscillations and mesial temporal lobe epilepsy. Neurosci Lett. (2018) 667:66–74. doi: 10.1016/j.neulet.2017.01.047, PMID: 28115239

[28] Jacobs J Staba R Asano E Otsubo H Wu JY Zijlmans M. High-frequency oscillations (HFOs) in clinical epilepsy. Prog Neurobiol. (2012) 98:302–15. doi: 10.1016/j.pneurobio.2012.03.001, PMID: 22480752 PMC3674884

[29] Zijlmans M Jiruska P Zelmann R Leijten FS Jefferys JG Gotman J. High-frequency oscillations as a new biomarker in epilepsy. Ann Neurol. (2012) 71:169–78. doi: 10.1002/ana.22548, PMID: 22367988 PMC3754947

[30] Matsumoto A Brinkmann BH Matthew Stead S Matsumoto J Kucewicz MT Marsh WR. Pathological and physiological high-frequency oscillations in focal human epilepsy. J Neurophysiol. (2013) 110:1958–64. doi: 10.1152/jn.00341.2013, PMID: 23926038 PMC3798937

[31] Cimbalnik J Brinkmann B Kremen V Jurak P Berry B Gompel JV. Physiological and pathological high frequency oscillations in focal epilepsy. Ann Clin Transl Neurol. (2018) 5:1062–76. doi: 10.1002/acn3.618, PMID: 30250863 PMC6144446

[32] Bragin A Engel J Jr Wilson CL Vizentin E Mathern GW. Electrophysiologic analysis of a chronic seizure model after unilateral hippocampal KA injection. Epilepsia. (1999) 40:1210–21. 10487183 10.1111/j.1528-1157.1999.tb00849.x

[33] Chen T Deng Y Sha L Shen Y Xu Q. A cynomolgus monkey model of temporal lobe epilepsy. Brain Res Bull. (2019) 144:187–93. doi: 10.1016/j.brainresbull.2018.11.001, PMID: 30423353

[34] Le Duigou C Bouilleret V Miles R. Epileptiform activities in slices of hippocampus from mice after intra-hippocampal injection of kainic acid. J Physiol. (2008) 586:4891–904. doi: 10.1113/jphysiol.2008.156281, PMID: 18755752 PMC2614071

[35] Felix B Leger ME Albe-Fessard D Marcilloux JC Rampin O Laplace JP. Stereotaxic atlas of the pig brain. Brain Res Bull. (1999) 49:1–137. doi: 10.1016/S0361-9230(99)00012-X, PMID: 10466025

[36] Van Nieuwenhuyse B Raedt R Sprengers M Dauwe I Gadeyne S Carrette E. The systemic kainic acid rat model of temporal lobe epilepsy: long-term EEG monitoring. Brain Res. (2015) 19:1–11. doi: 10.1016/j.brainres.2015.08.016 26381287

[37] Buzsaki G. Rhythms of the Brain. Oxford: Oxford University Press (2006).

[38] Delorme A Makeig S. EEGLAB: an open source toolbox for analysis of single-trial EEG dynamics including independent component analysis. J Neurosci Methods. (2004) 134:9–21. doi: 10.1016/j.jneumeth.2003.10.009 15102499

[39] Prerau MJ Brown RE Bianchi MT Ellenbogen JM Purdon PL. Sleep neurophysiological dynamics through the lens of multitaper spectral analysis. Physiology (Bethesda). (2017) 32:60–92. doi: 10.1152/physiol.00062.2015 27927806 PMC5343535

[40] Zhou Y You J Zhu F Bragin A Engel J Li L (Eds.) Automatic electrophysiological noise reduction and epileptic seizure detection for Stereoelectroencephalography. In *2021 43rd Annual International Conference of the IEEE Engineering in Medicine & Biology Society (EMBC)*; (2021): IEEE. 10.1109/EMBC46164.2021.9630651 34891250

[41] Li L Patel M Almajano J Engel J Jr Bragin A. Extrahippocampal high-frequency oscillations during epileptogenesis. Epilepsia. (2018) 59:e51–5. doi: 10.1111/epi.14041, PMID: 29508901 PMC6681898

[42] Navarrete M Alvarado-Rojas C Le Van QM Valderrama M. RIPPLELAB: a comprehensive application for the detection, analysis and classification of high frequency oscillations in electroencephalographic signals. PLoS One. (2016) 11:e0158276. doi: 10.1371/journal.pone.0158276, PMID: 27341033 PMC4920418

[43] Staba RJ Wilson CL Bragin A Fried I Engel J Jr. Quantitative analysis of high-frequency oscillations (80-500 Hz) recorded in human epileptic hippocampus and entorhinal cortex. J Neurophysiol. (2002) 88:1743–52. doi: 10.1152/jn.2002.88.4.1743, PMID: 12364503

[44] Shrout PE Fleiss JL. Intraclass correlations: uses in assessing rater reliability. Psychol Bull. (1979) 86:420–8. 18839484 10.1037//0033-2909.86.2.420

[45] McGraw KO Wong SP. Forming inferences about some intraclass correlation coefficients. Psychol Methods. (1996) 1:30–46. doi: 10.1037/1082-989X.1.1.30

[46] Weir JP. Quantifying test-retest reliability using the intraclass correlation coefficient and the SEM. J Strength Cond Res. (2005) 19:231–40. doi: 10.1519/00124278-200502000-00038, PMID: 15705040

[47] Li L Zeng L Lin Z-J Cazzell M Liu H. Tutorial on use of intraclass correlation coefficients for assessing intertest reliability and its application in functional near-infrared spectroscopy–based brain imaging. J Biomed Opt. (2015) 20:050801. doi: 10.1117/1.JBO.20.5.050801 25992845

[48] Atkinson G Nevill AM. Statistical methods for assessing measurement error (reliability) in variables relevant to sports medicine. Sports Med. (1998) 26:217–38. 9820922 10.2165/00007256-199826040-00002

[49] Borbély AA Baumann F Brandeis D Strauch I Lehmann D. Sleep deprivation: effect on sleep stages and EEG power density in man. Electroencephalogr Clin Neurophysiol. (1981) 51:483–95. doi: 10.1016/0013-4694(81)90225-X, PMID: 6165548

[50] Aboalayon KAI Faezipour M Almuhammadi WS Moslehpour S. Sleep stage classification using EEG signal analysis: a comprehensive survey and new investigation. Entropy. (2016) 18:272. doi: 10.3390/e18090272

[51] Terndrup TE Paskanik AM Fordyce WE Kanter RK. Development of a piglet model of status epilepticus: preliminary results. Ann Emerg Med. (1993) 22:164–70. doi: 10.1016/S0196-0644(05)80196-9, PMID: 8427425

[52] Terndrup TE Darnall R Knuth SL Bartlett D Jr. Effects of experimental cortical seizures on respiratory motor nerve activities in piglets. J Appl Physiol. (1985) 86:2052–8. doi: 10.1152/jappl.1999.86.6.2052 10368373

[53] Van Gompel JJ Bower MR Worrell GA Stead M Meier TR Goerss SJ. Swine model for translational research of invasive intracranial monitoring. Epilepsia. (2011) 52:e49–53. doi: 10.1111/j.1528-1167.2011.03096.x, PMID: 21627648 PMC3116097

[54] Silfverhuth MJ Kortelainen J Ruohonen J Suominen K Niinimaki J Sonkajarvi E. A characteristic time sequence of epileptic activity in EEG during dynamic penicillin-induced focal epilepsy–a preliminary study. Seizure. (2011) 20:513–9. doi: 10.1016/j.seizure.2011.03.006, PMID: 21511498

[55] Arabadzisz D Antal K Parpan F Emri Z Fritschy J-M. Epileptogenesis and chronic seizures in a mouse model of temporal lobe epilepsy are associated with distinct EEG patterns and selective neurochemical alterations in the contralateral hippocampus. Exp Neurol. (2005) 194:76–90. doi: 10.1016/j.expneurol.2005.01.029 15899245

[56] Carriero G Arcieri S Cattalini A Corsi L Gnatkovsky V De Curtis M. A Guinea pig model of mesial temporal lobe epilepsy following nonconvulsive status epilepticus induced by unilateral intrahippocampal injection of kainic acid. Epilepsia. (2012) 53:1917–27. doi: 10.1111/j.1528-1167.2012.03669.x, PMID: 22998690

[57] Raedt R Van Dycke A Van Melkebeke D De Smedt T Claeys P Wyckhuys T. Seizures in the intrahippocampal kainic acid epilepsy model: characterization using long-term video-EEG monitoring in the rat. Acta Neurol Scand. (2009) 119:293–303. doi: 10.1111/j.1600-0404.2008.01108.x, PMID: 19388152

[58] Schabus M Gruber G Parapatics S Sauter C Klösch G Anderer P. Sleep spindles and their significance for declarative memory consolidation. Sleep. (2004) 27:1479–85. doi: 10.1093/sleep/27.7.1479, PMID: 15683137

[59] Méndez M Radtke RA. Interactions between sleep and epilepsy. J Clin Neurophysiol. (2001) 18:106–27. doi: 10.1097/00004691-200103000-00003, PMID: 11435803

[60] Malow BA. Sleep deprivation and epilepsy. Epilepsy Curr. (2004) 4:193–5. doi: 10.1111/j.1535-7597.2004.04509.x, PMID: 16059497 PMC1176369

[61] Buzsaki G Silva FL. High frequency oscillations in the intact brain. Prog Neurobiol. (2012) 98:241–9. doi: 10.1016/j.pneurobio.2012.02.004, PMID: 22449727 PMC4895831

[62] Li L Kumar U You J Zhou Y Weiss SA Engel J. Spatial and temporal profile of high-frequency oscillations in posttraumatic epileptogenesis. Neurobiol Dis. (2021) 161:105544. doi: 10.1016/j.nbd.2021.105544 34742877 PMC9075674

[63] Kumar U Li L Bragin A Engel J Jr. Spike and wave discharges and fast ripples during posttraumatic epileptogenesis. Epilepsia. (2021) 62:1842–51. doi: 10.1111/epi.16958, PMID: 34155626 PMC8349888

[64] Bragin A Wilson C Engel J Jr. Chronic epileptogenesis requires development of a network of pathologically interconnected neuron clusters: a hypothesis. Epilepsia. (2000) 41:S144–52. doi: 10.1111/j.1528-1157.2000.tb01573.x, PMID: 10999536

[65] Engel J Jr Bragin A Staba R Mody I. High-frequency oscillations: what is normal and what is not? Epilepsia. (2009) 50:598–604. doi: 10.1111/j.1528-1167.2008.01917.x 19055491

[66] Jacobs J LeVan P Chander R Hall J Dubeau F Gotman J. Interictal high-frequency oscillations (80-500 Hz) are an indicator of seizure onset areas independent of spikes in the human epileptic brain. Epilepsia. (2008) 49:1893–907. doi: 10.1111/j.1528-1167.2008.01656.x, PMID: 18479382 PMC3792077

[67] Holmes GL Lenck-Santini PP. Role of interictal epileptiform abnormalities in cognitive impairment. Epilepsy Behav. (2006) 8:504–15. doi: 10.1016/j.yebeh.2005.11.014, PMID: 16540376

[68] Kleen JK Scott RC Holmes GL Roberts DW Rundle MM Testorf M. Hippocampal interictal epileptiform activity disrupts cognition in humans. Neurology. (2013) 81:18–24. doi: 10.1212/WNL.0b013e318297ee50, PMID: 23685931 PMC3770206

[69] Bragin A Engel J Jr Staba RJ. High-frequency oscillations in epileptic brain. Curr Opin Neurol. (2010) 23:151–6. doi: 10.1097/WCO.0b013e3283373ac8, PMID: 20160649 PMC4063284

[70] Carrera E Michel P Despland P-A Maeder-Ingvar M Ruffieux C Debatisse D. Continuous assessment of electrical epileptic activity in acute stroke. Neurology. (2006) 67:99–104. doi: 10.1212/01.wnl.0000223361.90278.ca, PMID: 16832085

[71] Sinkin M Kaimovsky I Komoltsev I Trifonov I Shtekleyn A Tsygankova M. Electroencephalography in the acute phase of stroke. Neurosci Behav Physiol. (2021):1–6. doi: 10.17116/jnevro202012008210

