# Supplementary material for: Intracranial electrophysiological recordings on a swine model of mesial temporal lobe epilepsy
Source: Front Neurol. 2023 Apr 17;14:1077702. doi: 10.3389/fneur.2023.1077702 (PMC10150775; doi:10.3389/fneur.2023.1077702)
Supplement: Supplementary file 1 [file Table_1_v1.DOCX]

Table S1. Individual weekly HFO rates (num/min) for intraclass correlation coefficient analysis

|  | **week1** | **week2** | **week3** | **week4** |
| --- | --- | --- | --- | --- |
| **101** | 4.27 | 5.25 | 1.71 | 3.25 |
| **102** | 7.09 | 10.25 | 4.35 | 7.25 |
| **103** | 4.57 | 7.25 | 3.77 | 5.25 |
| **104** | 2.89 | 6.5 | 2.22 | 4.75 |
| **110** | 4.25 | 4 | 2.58 | 3 |
| **111** | 4.99 | 6.75 | 1.26 | 3.25 |
| **112** | 4.24 | 6 | 2.89 | 4.5 |
| **113** | 2.63 | 6.25 | 4.27 | 2.75 |
| **105** | 1.5 | 1.25 | 1.83 | 3 |
| **109** | 1.83 | 2 | 1.15 | 1 |
| **114** | 1.91 | 2.5 | 2.16 | 3 |
| **115** | 1.5 | 0.75 | 1.71 | 1.75 |
