# Supplementary figures and images for: Intracranial electrophysiological recordings on a swine model of mesial temporal lobe epilepsy
Source: Front Neurol. 2023 Apr 17;14:1077702. doi: 10.3389/fneur.2023.1077702 (PMC10150775; doi:10.3389/fneur.2023.1077702)

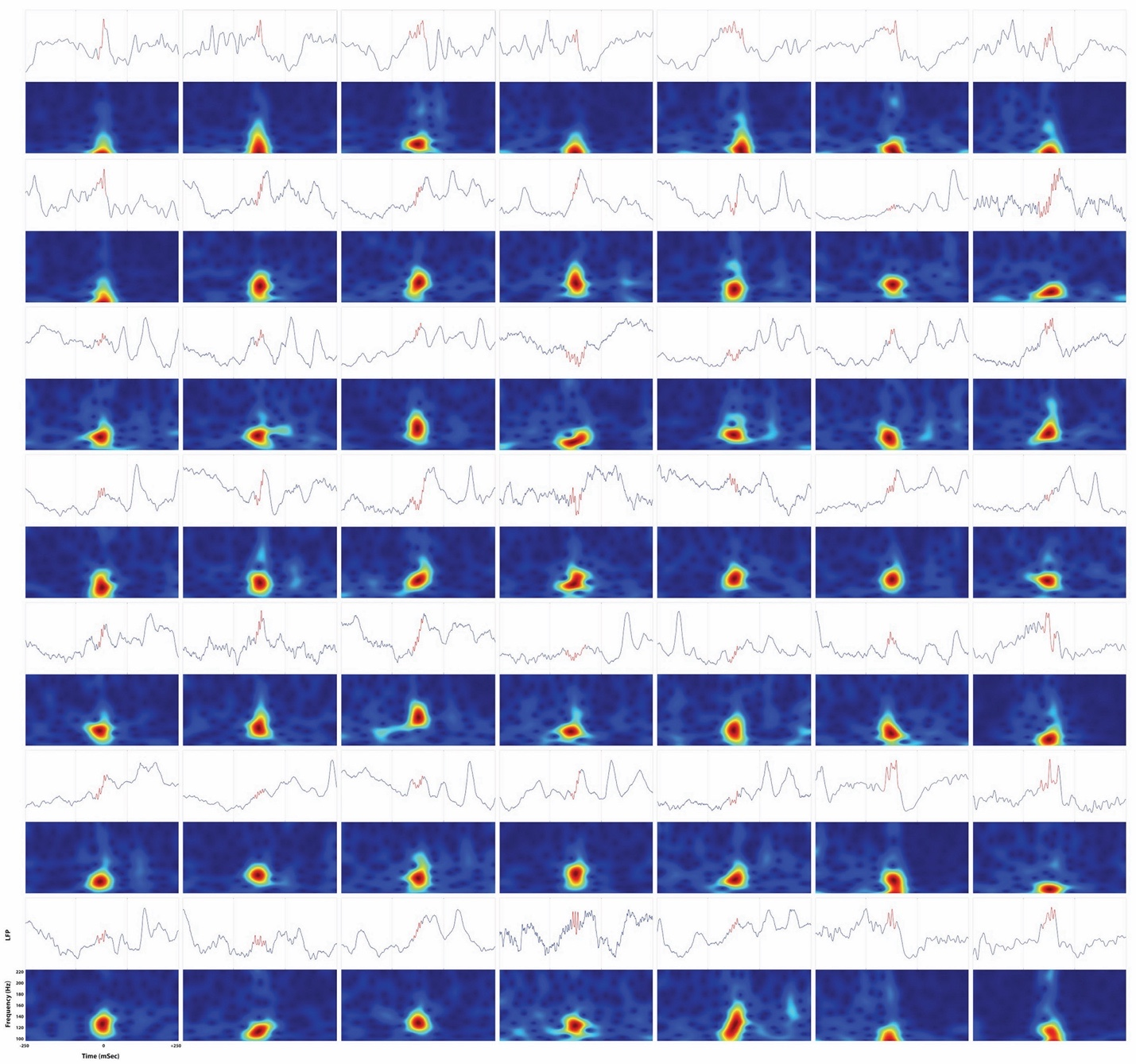

Supplement: Supplementary file 2 [file Image_1_v1.TIF]
